# Identification of BACE2 as an avid ß-amyloid-degrading protease

**DOI:** 10.1186/1750-1326-7-46

**Published:** 2012-09-17

**Authors:** Samer O Abdul-Hay, Tomoko Sahara, Melinda McBride, Dongcheul Kang, Malcolm A Leissring

**Affiliations:** 1 Department of Neuroscience, Mayo Clinic, 4500 San Pablo Road, Birdsall Bldg., Rm. 117, Jacksonville, FL, 32224, USA

**Keywords:** Amyloid-ß-protein, Alzheimer disease, ß-site APP-cleaving enzyme-1, ß-site APP-cleaving enzyme-2, Functional screen, Gene therapy, Protease, Proteolytic degradation

## Abstract

**Background:**

Proteases that degrade the amyloid ß-protein (Aß) have emerged as key players in the etiology and potential treatment of Alzheimer’s disease (AD), but it is unlikely that all such proteases have been identified. To discover new Aß-degrading proteases (AßDPs), we conducted an unbiased, genome-scale, functional cDNA screen designed to identify proteases capable of lowering net Aß levels produced by cells, which were subsequently characterized for Aß-degrading activity using an array of downstream assays.

**Results:**

The top hit emerging from the screen was ß-site amyloid precursor protein-cleaving enzyme 2 (BACE2), a rather unexpected finding given the well-established role of its close homolog, BACE1, in the production of Aß. BACE2 is known to be capable of lowering Aß levels via non-amyloidogenic processing of APP. However, in vitro, BACE2 was also found to be a particularly avid AßDP, with a catalytic efficiency exceeding all known AßDPs except insulin-degrading enzyme (IDE). BACE1 was also found to degrade Aß, albeit ~150-fold less efficiently than BACE2. Aß is cleaved by BACE2 at three peptide bonds—Phe19-Phe20, Phe20-Ala21, and Leu34-Met35—with the latter cleavage site being the initial and principal one. BACE2 overexpression in cultured cells was found to lower net Aß levels to a greater extent than multiple, well-established AßDPs, including neprilysin (NEP) and endothelin-converting enzyme-1 (ECE1), while showing comparable effectiveness to IDE.

**Conclusions:**

This study identifies a new functional role for BACE2 as a potent AßDP. Based on its high catalytic efficiency, its ability to degrade Aß intracellularly, and other characteristics, BACE2 represents a particulary strong therapeutic candidate for the treatment or prevention of AD.

## Background

Alzheimer disease (AD) is a progressive and presently incurable neurodegenerative disorder characterized by abnormal accumulation of the amyloid β-protein (Aβ) in brain regions important for mnemonic and cognitive functions. Aß is a heterogeneous mixture of peptides ranging from 37 to 43 amino acids in length [1] produced via sequential cleavage of the amyloid precursor protein (APP) by BACE1 and the presenilin/γ-secretase complex [2-4]. Autosomal-dominant mutations in 3 genes—APP and presenilin-1 and −2—are known to cause rare, familial forms of AD either by increasing the production of all forms of Aß or by increasing the relative production of longer, more amyloidogenic forms, such as Aß42 [5]. Nevertheless, the precise mechanisms underlying sporadic AD, which makes up the vast majority of cases, remain to be elucidated.

Aß-degrading proteases (AßDPs) are potent regulators of cerebral Aß levels and, as such, represent important players in the etiology and potential treatment of AD [6]. Amyloidogenesis and downstream cytopathology can be attenuated and even completely prevented by enhancing the activity of any of several AßDPs, while, conversely, genetic deletion of one or more AßDPs leads to significant elevations in cerebral Aß [7]. Significantly, patients with sporadic AD were recently shown to exhibit defects in the clearance of Aß (rather than increases in its production) [8] and, in light of the large body of evidence implicating AßDPs in the regulation of cerebral Aß levels [7], it is reasonable to infer that defects in one or more AßDPs could contribute to impaired Aß clearance. While more than twenty proteases are now known to degrade Aß [7], these were not identified through any systematic approach, but instead emerged haphazardously from a disconnected set of largely serendipitous discoveries. Nevertheless, essentially all AßDPs now known to regulate Aß in vivo were originally identified through exclusively in vitro or cell-based approaches [9].

To discover new AßDPs more systematically, we conducted an unbiased, cell-based, functional screen of 352 proteases in the human genome. The top Aß-lowering protease emerging from this screen was ß-site APP-cleaving enzyme-2 (BACE2) [10]. Previous studies have shown that BACE2 can lower Aß levels via α-secretase-like cleavage of APP within the Aß sequence [11-16], an activity that has been dubbed “θ-secretase” [17]. However, we found that BACE2 is also a remarkably avid AßDP, with a catalytic efficiency exceeding all other known AßDPs except insulin-degrading enzyme (IDE).

## Results and discussion

### Functional screen for novel AßDPs

**Figure 1:**
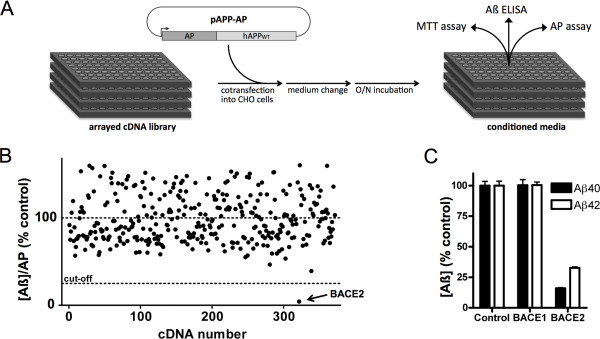
**Overview and outcome of functional screening for novel Aß-lowering proteases. *A***, Cartoon illustrating the overall design of the screen. Briefly, an arrayed collection of 352 protease-encoding cDNAs was cotransfected into CHO cells together with an APP-AP fusion construct. Following a medium change and overnight incubation, Aß40 levels, AP activity, and cytotoxicity (via MTT assay) were analyized in the resulting conditioned media. ***B***, Results of the screen, expressed as [Aß40]/AP ratios normalized to intraplate controls. Data are mean of 4 replicates. Note that the largest decrease in Aß levels by far was acheived by BACE2. ***C***, Confirmation of the results of the screen. Following scale-up and sequence verification, cDNAs encoding BACE2 and its homolog, BACE1, were cotransfected together with APP-AP into CHO cells. Consistent with the outcome of the medium-throughput screen, BACE2, but not BACE1, expression resulted in significant decreases in the levels of both Aß40 and Aß42. Data are mean ± SEM of 4 replicates, and are normalized to vector-only controls.

To identify novel AßDPs, we performed a cell-based functional screen using a commercial library consisting of 352 full-length, sequence-verified, human cDNAs encoding diverse members of all protease classes. We experimented with several approaches before settling on a final configuration for the primary screen. Assays designed to monitor degradation of exogenous Aß were found to be confounded by the highly dominant effect of IDE, which mediates the vast majority of extracellular Aß degradation in cultured cells [18-20]. Transient transfection of cDNAs into cell lines stably expressing APP was also tried, but this approach suffered from incomplete transfection efficiency, which attenuated the effect on net extracellular Aß levels. We therefore elected to conduct the screen by co-transfecting protease-encoding cDNAs, together with positive and negative controls, into a rodent cell line (CHO cells) together with a plasmid encoding wild-type human APP fused to alkaline phosphatase (AP) (see Figure 1A; *Methods*). Use of the APP-AP construct ensured that human Aβ production was limited to cells also expressing candidate AßDPs, while also providing an internal control for transfection efficiency (via AP activity). Importantly, the co-transfection strategy also increased the likelihood of detecting AßDPs that degrade Aß intracellularly, prior to its secretion, in addition to those that act exclusively extracellularly. Cytotoxicity was also quantified via an MTT conversion assay, but no significant cell death was detected so these data were not incorporated into subsequent analyses. The screen was performed in quadruplicate and, for each well, the ratio of Aß40 concentration to AP activity was calculated, then normalized to appropriate intra-plate controls (Figure 1B).

From among the 352 proteases examined, by far the largest decrease in normalized Aß levels (97 ± 1.2%) was induced by BACE2, which was in fact the only protease to lower Aß levels more than 75%, our pre-determined cut-off for viable hits (Figure 1B).

### BACE2 transfection lowers Aβ levels

To confirm and extend the results obtained in the cDNA screen, we compared the degree to which overexpression of BACE2 and its homolog BACE1 [21] affected the net production of different Aß species. Consistent with the results of the primary screen, BACE2 transfection in CHO cells decreased the levels of both Aβ40 and Aβ42 (Figure 1C). Overexpression of BACE1 in this cell type, by contrast, had no effect on net Aß levels (Figure 1C). We note that BACE1 overexpression would not be expected to increase Aß production in CHO cells, since previous studies have established that γ-secretase, rather than ß-secretase, is the rate-limiting step in Aß production in this cell type [22].

### BACE2 and BACE1 degrade Aß in vitro

**Figure 2:**
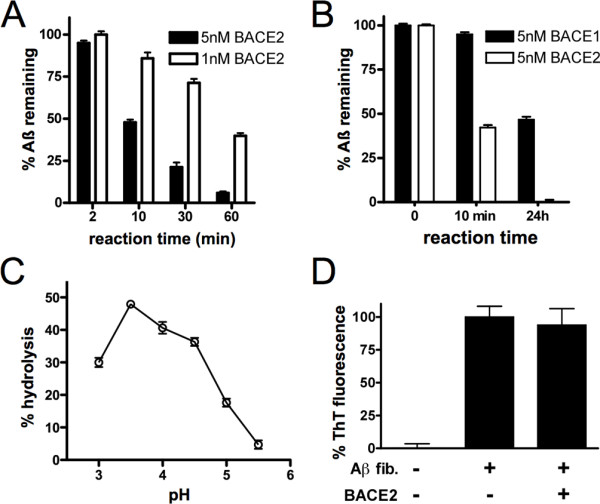
**BACE2 degrades Aß in vitro. *A***, Percent Aß remaining following incubation with different concentrations of recombinant BACE2 for various lengths of time. Data are mean ± SEM of 4 replicates, normalized to protease-free controls. ***B***, Comparison the relative Aß-degrading ability of recombinant BACE2 vs. BACE1. Note that 24 h incubation with BACE1 was required to achieve approximately the same extent of degradation as effected by BACE2 in 10 min. Data are mean ± SEM of 3 replicates, normalized to protease-free controls. ***C***, BACE2 activity is pH dependent. Percent Aß degradation catalyzed by equivalent amounts of BACE2 at different pH values. Data are mean ± SEM of 4 replicates. ***D***, BACE2 does not degrade fibrillar Aß. Lack of effect of BACE2 (10 nM) on preformed Aß42 fibrils following incubation at 37°C for 5 d, as determined by thioflavin T fluorescence. Data are mean ± SEM of 3 replicates.

Expression of BACE2 in cells could lower Aß levels either directly, via proteolytic degradation, or indirectly, via alternative mechanisms such as hydrolysis of APP or APP C*-*terminal fragments (CTFs) [11-16]. To distinguish these possibilities, we tested the ability of recombinant BACE2 to hydrolyze synthetic Aß in vitro, using a well-established fluorescence polarization-based Aß degradation assay [23]. Recombinant BACE2 was found to avidly degrade Aß in this paradigm, confirming that BACE2 is indeed a bona fide AßDP (Figure 2A). Recombinant BACE1 also hydrolyzed Aß, indicating that it too is an AßDP (Figure 2B). However, BACE1 was much less efficient than BACE2, requiring 24 h to degrade Aß to a similar extent as was achieved following a 10-min incubation with BACE2 (Figure 2B). Based on these results, the efficiency of BACE1 would appear to be ~150-fold lower than that of BACE2.

### BACE2-mediated Aß degradation is pH-dependent

As an aspartyl protease, the catalytic efficiency of BACE2 is expected to be pH-dependent. To confirm this, we compared the rate of hydrolysis of Aß40 across a range a pH values. Consistent with expectations, BACE2 was found to be maximally effective at pH 3.5 (Figure 2C), and decreasingly effective at higher pH values. These findings strongly suggest that BACE2 would not be operative at the cell surface or within the extracellular space.

### BACE2 does not degrade fibrillar Aß

Individual AßDPs can be categorized in terms of their ability or inability to degrade fibrillar forms of Aß. Many well-established AßDPs, such as IDE and NEP, avidly degrade monomeric Aß but cannot degrade fibrillar forms and are therefore categorized as pure peptidases. Others, such as plasmin, degrade Aß fibrils and thus can also be categorized as fibrilases [7]. To determine to which category BACE2 belongs, we incubated recombinant BACE2 with pre-formed fibrils of Aß42 and quantified the degree of aggregation by thioflavin T fluorescence. No significant reduction in aggregation was observed, even following incubation at 37°C for up to 3 d (Figure 2D). These results suggest that, as is true for the majority of AßDPs [7], BACE2 does not degrade Aß fibrils.

### BACE2 cleaves Aß at 3 sites

**Figure 3:**
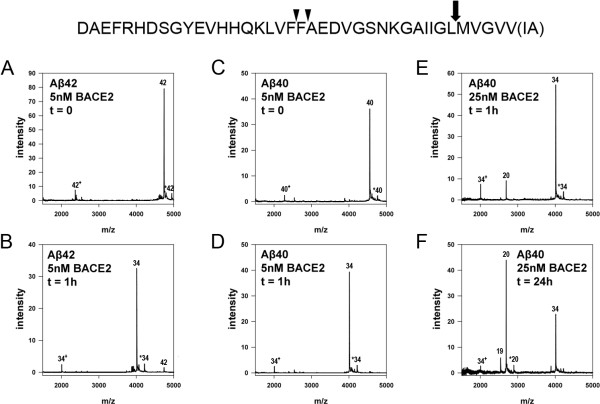
**Determination of peptide bonds within Aß hydrolyzed by BACE2. ***Top*, Summary of cleavage sites determined from data in **AF**, showing the major site (*block arrow*) and two minor sites (*arrow heads*). At t = 0 (**A**, **C**), intact Aß42 (**A**) and Aß40 (**C**) represent the only species present. Following incubation of Aß42 and Aß40 with 5nM BACE2 for 1 h (**BD**, respectively), the full-length Aß species are essentially completely absent and replaced by Aß34. ***E****,****F***, Additional Aß cleavage products are produced following incubation with larger amounts of BACE2 (25 nM) for longer lengths of time. By 1 h (**E**), a new peak corresponding to Aß20 is produced. By 24 h (**F**), Aß20 becomes the major species present, and Aß19 is also produced. Double-charged fragments are denoted by “+”, and “*” represents the modification of a fragment by AEBSF, which leads to a 183-Da increase in MW, as previously reported [46].

We next investigated which peptide bond(s) within Aβ are hydrolyzed by BACE2 and BACE1. To that end, we co-incubated N-terminally biotinylated Aβ40 or Aβ42 (300nM) with BACE2 (5 nM) and analyzed the products by immunoprecipitation/mass spectrometry (IP/MS) (see *Methods*). Within 1 h, BACE2 almost completely hydrolyzed both Aß species, generating the shorter fragment, Aβ34, in both cases (Figure 3A-D). To test whether any additional cleavages can occur, we incubated N-terminally biotinylated Aβ40 (300 nM) with a larger amount of BACE2 (25 nM) for 1 and 24 h. At these higher concentrations and longer incubation times, Aβ19 and Aβ20 were the principal N-terminal fragments remaining at the end of the reaction (Figure 3E-F). Collectively, these in vitro results suggest that BACE2 cleaves Aβ at three different positions: Phe19-Phe20, Phe20-Ala21, and Leu34-Met35, with the latter cleavage site being the initial and principal one, as is consistent with previous observations [13,14,24].

**Figure 4:**
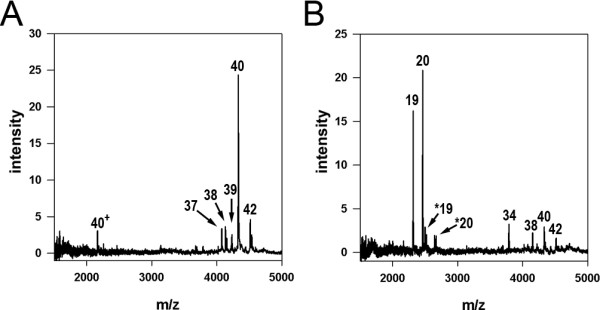
**Overexpression of BACE2 in cells yields Aß fragments identical to those produced in vitro. AB**, Spectra of Aß fragments determined by IP/MS analysis of the conditioned media of CHO cells transfected with APP and empty vector (**A**) or APP and BACE2 (**B**) (see *Methods***).*A***, APP expression alone produces peaks corresponding to Aß42, Aß40, Aß39, Aß38 and Aß37. ***B***, Co-expression of APP and BACE2 results in decreases in the relative abundance of the aforementioned Aß species and the appearance of three new fragments: Aß34, Aß20 and Aß19. Double-charged fragments are denoted by “+”, and “*” represents the modification of a fragment by AEBSF, which leads to a 183-Da increase in MW, as previously reported [46].

To confirm whether BACE2 cleaves Aβ at the same sites in a more physiological setting, we analyzed Aß species in the conditioned media of cells expressing APP-AP either alone or together with BACE2 by IP/MS (see *Methods*). As expected for cells expressing APP-AP alone, the medium from these cells contained Aβ42, Aβ40, Aβ39, Aβ38, and Aβ37 (Figure 4A). BACE2 expression suppressed the signal of all of these species, and new peaks corresponding to Aβ19, Aβ20, and Aβ34 emerged (Figure 4B), confirming that the cleavage sites mediated by BACE2 in vitro are also hydrolyzed in intact cells. The appearance of Aß34 is particularly notable, because cleavage at position 34 can only occur after production of full-length Aß, as this peptide bond is positioned within the transmembrane domain of APP, as has been shown previously [24]. Although this result clearly indicates that BACE2 does indeed degrade Aß after it is produced, it is not possible to quantify the extent to which the Aß19 and Aß20 peaks are the result of θ-secretase activity or subsequent degradation of the Aß34 fragment (or full-length Aß). As a consequence, it is difficult to estimate the exact extent to which the Aß-lowering effect of BACE2 can be assigned to non-amyloidogenic processing versus Aß degradation per se in experimental paradigms of this type.

### BACE2 degrades Aß more efficiently than well-established AßDPs

**Figure 5:**
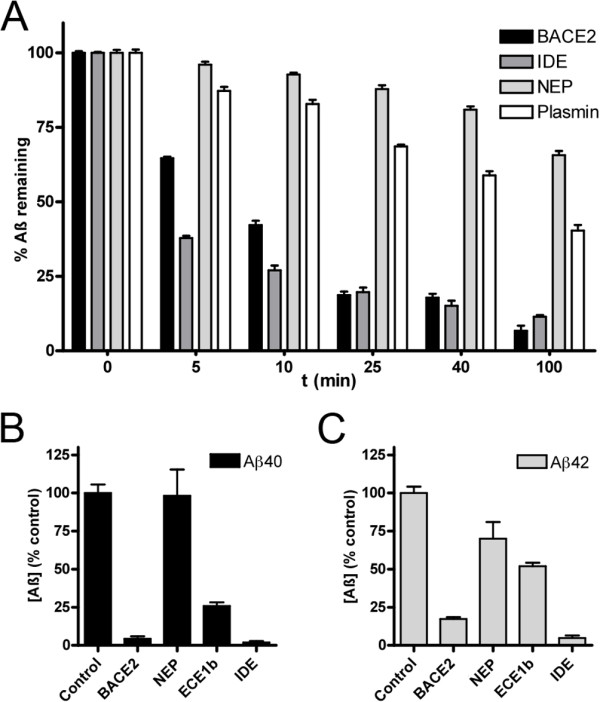
**Comparison of the efficacy of BACE2 relative to other well-established AßDPs in vitro and in cultured cells. *A***, Degradation of Aß in vitro by equivalent nominal concentrations (5 nM) of recombinant BACE2, IDE, NEP and plasmin. Note that BACE2 degrades Aß at a faster rate than NEP and plasmin, but not IDE. ***B***,***C***, Effects on Aß40 (**A**) and Aß42 (**C**) levels following cotransfection of CHO cells with APP together with equivalent quantities of cDNAs encoding BACE2, ECE1b and IDE. In good agreement with the results in vitro (**A**), BACE2 lowers the levels of both Aß species to an extent exceeding NEP and ECE1b, but comparable to IDE. Data are mean ± SEM of 4 replications, normalized to controls cotransfected with empty vector (V_o_).

Having established BACE2 as an AßDP, we next investigated how BACE2 compares to other known AßDPs in terms the ability to degrade Aß in vitro and to lower net Aß levels in cells. To compare the relative efficiency of BACE2 in vitro, we monitored the degradation of a fixed amount of Aß (200 nM) by recombinant BACE2 (5 nM) as compared to equal quantities of several well-established AßDPs, including IDE, NEP and plasmin. Under these conditions, BACE2 hydrolyzed Aß more efficiently than all other AßDPs except IDE (Figure 5A). We note that the concentration of Aß used in this experiment was considerably lower than the *K*_M_ for each of the proteases tested (see [23] and below), making the initial velocity of this reaction a good index of the relative catalytic efficiency.

### Kinetics of Aß degradation by BACE2

To investigate the catalytic efficiency of BACE2 more quantititatively, we determined the kinetics of degradation of both Aß40 and Aß42 by BACE2 (see *Methods*). For this analysis, we were careful to use freshly prepared batches of monomeric human Aβ40 and Aβ42 peptides, which we routinely prepare by size-exclusion chromatography and which have been extensively characterized [25,26]. BACE2 cleaved both Aß species with similar kinetics, exhibiting apparent *K*_M_ values in the low micromolar range and albeit with apparent *k*_cat_ values slightly higher for Aß40 relative to Aß42 (0.135 ± 0.016 min^-1^ and 0.025 ± 0.005 min^-1^, respectively; Table 1). In terms of catalytic efficiency (*k*_cat_/*K*_M_), BACE2 degrades Aβ40 approximately 4-fold more efficiently than Aβ42 (Table 1). These parameters exceed the published values for most other well-characterized AßDPs, including NEP [23], ECE1 [27], and plasmin [23], while being comparable to those of IDE [23,28]. Consequently, these values are in good agreement with the side-by-side comparison of Aß degradation in vitro discussed above (Figure 5A).

**Table 1:** Kinetic parameters of Aß40 and Aß42 degradation by BACE2

	**Aß40**	**Aß42**
***K***_**M**_**(μM)**	2.8 ± 0.7	2.3 ± 0.6
**V**_**max**_**(μM min**^**-1**^**)**	0.68 ± .083	0.12 ± 0.025
***k***_**cat**_**(min**^**-1**^**)**	0.135 ± 0.016	0.025 ± 0.005
***k***_**cat**_**/*K***_**M**_**(M**^**-1**^ **min**^**-1**^**)**	4.82 x 10^7^	1.07 x 10^7^

To investigate the relative ability of BACE2 to lower Aß levels under more physiological conditions, we co-transfected CHO cells with APP together with BACE2 or several other AßDPs, then quantified net Aß40 and Aß42 levels in the conditioned medium by ELISA. We emphasize that this approach cannot control for intrinsic differences in transcription or translation efficiency, and, in the case of BACE 2, the Aß-lowering effect can also be mediated to an undetermined degree by BACE2-mediated θ-secretase activity. Nevertheless, the results were in good agreement with the in vitro findings: BACE2 lowered net Aß40 and Aß42 levels to a comparable extent as IDE, with both of the latter being significantly more effective than NEP or plasmin (Figure 5B, C).

### BACE2 colocalizes with Aβ intracellularly

**Figure 6:**
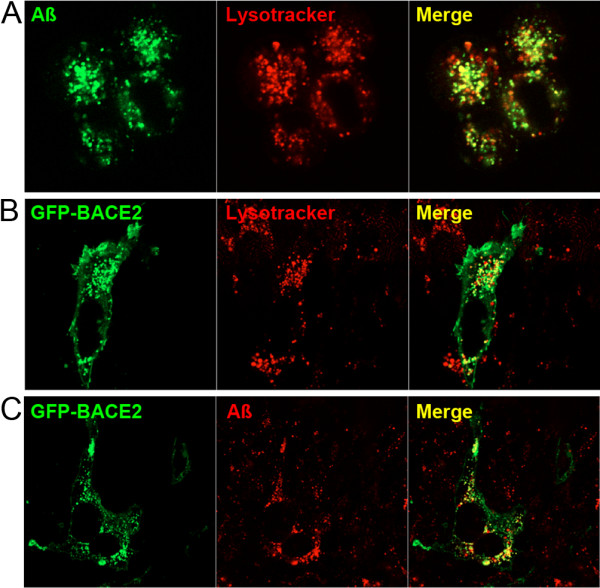
**BACE2 is localized to intracellular compartments relevant to Aß degradation. *A***, Exogenous administration of fluorescently labeled Aß40 (*green*) to CHO cells results in accumulation at intracellular sites overlapping with lysosomes, as labelled by Lysotracker Red (*red*) and visualized by confocal microscopy. ***B***, BACE2 is expressed in multiple intracellular compartments, including lysosomes. Distribution of GFP-tagged BACE2 (*green*) in cells labeled with Lysotracker Red (*red*) shows significant localization within lysosomes (*yellow*). ***C***, BACE2 colocalizes with exogenously administered Aß. Confocal images showing significant overlap (*yellow*) between BACE2 (*green*) and fluorescently labeled Aß (*red*). For these experiments, cells were imaged within 5 minutes of washing in cold PBS to remove medium containing excess fluorescently labeled Aß. Note that the the majority of BACE2-GFP-expressing cells contianed very low levels of fluorescent Aß (see Figure 7), and the particular cell shown exhibited relatively high levels of internalized Aß, allowing us to highlight the overlap with BACE2.

Having determined that BACE2 is functionally among the most efficient AßDPs yet discovered, we subsequently investigated the subcellular localization of BACE2, focusing in particular on the extent to which it colocalizes with Aβ in acidic compartments, where BACE2 is expected to be operative. In agreement with other published findings [29], application of fluorescently tagged Aß to live cells resulted in its accumulation at intracellular sites largely overlapping with lysosomes (Figure 6A). To test whether BACE2 is also localized to lysosomes and/or other compartments containing Aß, we analyzed CHO cells expressing BACE2 tagged at its N-terminus with green fluorescent protein (BACE2-GFP). As determined by confocal microscopy, BACE2-GFP was found to be present in lysosomes (Figure 6B) and also to overlap significantly with fluorescently labeled Aß (Figure 6C).

### BACE2 degrades Aß at intracellular sites

**Figure 7:**
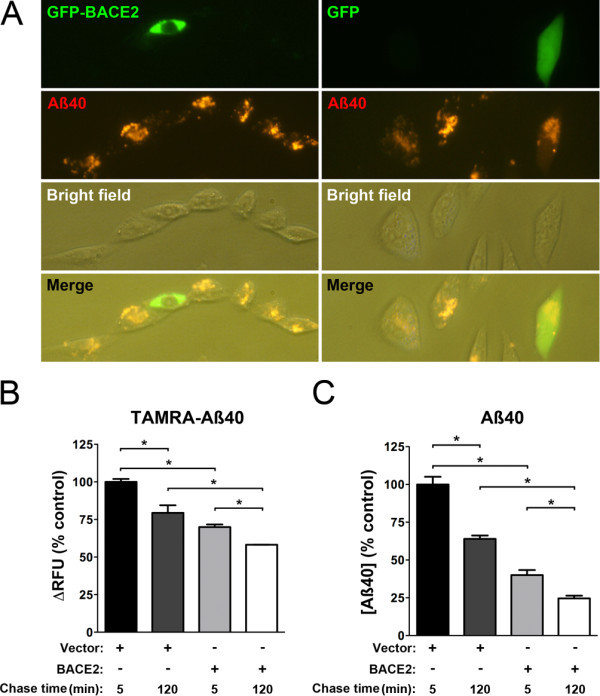
**BACE2 degrades Aß at intracellular sites. *A***, CHO cells expressing GFP-BACE2 (*green, left*), but not those expressing GFP alone (*green, right*), exhibit marked reductions in intracellular Aß (*red*). For these experiments, cells were loaded for 6 h with 400 nM fluorescently labeled Aß40, washed, then incubated at 37°C for 1 h prior to imaging by conventional fluorescence microscopy. ***B***,***C***, BACE2 overexpression significantly lowers intracellular Aß. ***B***, Quantification of intracellular pools of fluorescently labeled Aß40 in CHO cells 0 and 2 hours after loading. ***C***, Relative levels of intracellular (unmodified) Aß40 in CHO cells 0 and 2 hours after loading, as quantified by ELISA. Data are mean ± SEM of 3 replicates, normalized to vector-only controls. *P <0.05 by Tukey’s multiple comparisons test.

To directly assess whether BACE2 degrades Aß at intracellular sites, we tested the ability of BACE2-expressing cells to degrade exogenously applied Aß by multiple methods. Cells overexpressing BACE2-GFP and loaded with fluorescently tagged Aß40 showed significantly reduced intracellular Aß 1 h after washing, but this was not the case for cells overexpressing GFP alone (Figure 7A). Consistent with this, levels of intracellular Aß, both fluorescently tagged and unmodified, were found to be consistently lower in cells overexpressing (untagged) BACE2 relative to vector-trasfected controls (Figure 7B,C). Notably, significantly lower levels of intracellular Aß were observed both 5 min and 2 h after washing in multiple paradigms. Collectively, these results strongly suggest that BACE2 is a bona fide AßDP that avidly degrades Aß within acidic compartments.

## Conclusions

One of the most fruitful outcomes of the genomic revolution is the emergence of genome-scale collections of full-length, sequence verified cDNAs. Combined with appropriate functional assays, cDNA libraries have catalyzed significant advances in our understanding of AD pathogenesis, including the seminal discovery that ß-secretase activity, the first step in the production of Aß, is mediated by BACE1 [21]. Here, we utilized a similar approach to discover new candidate AßDPs, using a functional assay sensitive to both extracellular and intracellular Aß degradation (as well as other potential Aß-lowering mechanisms). Rather unexpectedly, the top hit emerging from a screen of 352 proteases was BACE2, a close homolog of BACE1. Subsequent characterization confirmed that, in addition to BACE2’s established ability to lower Aß production via θ-secretase-mediated processing of APP [11-16], BACE2 also avidly degrades Aß with a catalytic efficiency exceeding almost all well-established AßDPs.

The finding that BACE2 is an avid AßDP suggests a novel and unexpected role for this protease in the pathogenesis of AD. Indeed, given its close homology with BACE1, it was initially hypothesized that BACE2 might mediate the *production* of Aß, via β-secretase cleavage of APP, instead [15,16]. However, most evidence now suggests that BACE2 does not contribute appreciably to Aß production in vivo [3]. For instance, cultured neurons from BACE2 knockout mice did not show reductions in Aß following transfection with APP [30] and conversely, overexpression of BACE2 in APP transgenic mice failed to increase cerebral Aß levels, as would be expected if BACE2 possessed ß-secretase-like activity.

In addition to its potent ability to degrade Aß, BACE2 also possesses a second Aß-lowering function for BACE2, one that is quite independent of Aß degradation. Specifically, BACE2 has been shown to cleave APP and the ß-secretase-derived APP-CTF within the Aß sequence, in a manner analogous to α-secretase [11-16]. This activity, dubbed θ-secretase [17], occurs at positions 19 and 20 within the Aß sequence, precisely the same cleavage sites identified in the present study [13,14]. As is true for α-secretase, θ-secretase activity lowers Aß levels by shuttling APP away from the amyloidogenic processing pathway [11-16].

As confirmed by previous work [24], we found that BACE2 also cleaves Aß at the Leu34-Met35 peptide bond, which was in fact the initial and principal site of cleavage. Notably, cleavage at this position can only occur after production of full length Aß by ß- and γ-secretase, because this peptide bond in APP or in APP CTFs is normally embedded within the cell membrane [24]. This fact, together with the finding that Aß34 is produced in cells overexpressing of BACE2 and APP, provides clear evidence that the Aß-degrading activity of BACE2 contributes significantly to the overall Aß-lowering effect of BACE2 overexpression, even in the context of concurrent θ-secretase activity.

Given that BACE2 can lower Aß both by decreasing its production and by mediating its degradation, which of these mechanisms are relevant to the pathogenesis or the potential treatment of AD? The answer depends critically on precisely where and to what extent BACE2 is expressed in vivo. Although BACE2 protein is readily detected in brain extracts [15,30-36], and its activity has even been shown to be comparable to that of BACE1 in post-mortem brain [31,33], there is conflicting evidence about which cell types express BACE2. Studies in mice, on the one hand, suggest that the protease is expressed abundantly in glia but only minimally in neurons [30]. To the extent that these findings apply to humans, θ-secretase cleavage of APP by BACE2 would be unlikely to play any significant pathophysiological role in AD, given that APP itself is expressed predominantly in neurons, with only modest expression levels in non-neuronal brain cells [31]. On the other hand, multiple studies in post-mortem human brain tissue have reported detectable BACE2 expression not only in astrocytes, but also in neurons [15,33], suggesting that the θ-secretase activity of BACE2 may, to some extent, contribute to the overall economy of brain Aß. The pathophysiological relevance of BACE2’s function as an AßDP is similarly difficult to predict and likewise dependent on the extent to which the protease is expressed in neurons. Astrocytes are known mediate the clearance of Aß [37], but the contribution of intra-astrocytic Aß degradation relative to intraneuronal or extracellular degradation in vivo remains to be established. As was true for other AßDPs first identified in cells [9], the answer to these questions will require further study in relevant animal models.

Notwithstanding uncertainty about its role in AD pathogenesis, a number of considerations suggest that BACE2 represents an especially strong therapeutic candidate, particularly for gene therapy-based approaches. BACE2 can lower Aß catalytically via two independent mechanisms, and its Aß-degrading ability alone exceeds that of most other AßDPs, some of which are being considered for gene therapy clinical trials [38]. Moreover, as an aspartyl protease, BACE2 possesses distinct advantages relative to other AßDPs. First, it is operative with subcellular compartments most relevant to Aß production—i.e., those containing active ß- and γ-secretase, which are both aspartyl proteases—thus allowing it to impact Aß levels prior to secretion. In this connection, there is growing evidence that intracellular Aß may represent an especially pathogenic role in AD [39], so modulation of this pool may be particularly appropriate therapeutically. Second, because BACE2 is operative exclusively at intracellular sites, its expression could be readily restricted to the site of administration. This is in contrast to many other AßDPs which are secreted and/or active extracellularly [19,40,41] and thus less capable of being confined to specific regions.

In conclusion, this study identifies BACE2 as a novel and highly efficient AßDP. This newly identified function of BACE2, together with its established ability to also lower Aß production via θ-secretase activity, suggests that BACE2 may play a significant role in AD pathogenesis. Moreover, even if BACE2 plays no role in the etiology of AD, BACE2 nevertheless represents a particularly attractive candidate for gene therapeutic approaches to the treatment of prevention of this presently incurable disease.

## Methods

### cDNA screening

A library of 352 full-length, sequence verified, human cDNAs encoding diverse members of all protease classes was purchase from a commercial source (OriGene Technologies, Inc.) in 96-well format (100 ng/well). For negative and positive controls, a subset of blank wells on each plate were supplemented with empty vector or a construct expressing a well-established AßDP, human ECE1b [27], respectively (100 ng/well). As a source of human Aß and also as a transfection control, each well was cotransfected with a hybrid construct, APP-AP (60 ng/well), comprised of a vector expressing wild-type human APP fused at its N-terminus with alkaline phosphatase (AP) [42]. Additional blank wells were left untreated for cell-free background controls. CHO cells (4.8 x 10^4^/well) suspended in DMEM/Opti-MEM supplemented with 5%FBS were then co-transfected with APP-AP and protease-encoding cDNAs using Fugene 6.0, according to manufacturer’s recommendations (Promega Corp.). Transfected cells were allowed to grow overnight under standard cell culture conditions (5% CO_2_; 37°C; 95% humidity) then the medium was exchanged. 24 h later, the conditioned media were collected for downstream analysis (see below). All experiments were conducted in compliance with and with approval by the Mayo Clinic Institutional Review Board.

### AP activity

Following heat treatment to inactivate endogenous phosphatases (65°C for 15 min) present in the media, conditioned media (30 μL/well) was added to 96-well plates containing AP substrate, 4-nitrophenylphosphate (170 μL/well, 2 mg/mL), dissolved in AP buffer (1 M diethanolamine, 0.5 mM MgCl_2_, 10 mM L-homoarginine, pH 9.8). Plates were incubated for 30 min and AP activity was determined from absorbance (OD_405_) using a SpectraMax® M5^e^ multilabel plate reader (Molecular Devices).

### Aβ ELISA

Aβ levels were quantified using a sandwich ELISA system based on antibody pairs 33.1.1/13.1.1 for Aβ40 and 2.1.3/4 G8 for Aβ42 as described previously [43]. Conditioned media were supplemented with Complete^TM^ Protease Inhibitor Cocktail (Roche) just after collection and analyzed immediately. For experiments quantifying intracellular Aß, cells were plated in in 96-well plates (2 x 10^4^ cells per well) and transfected with BACE2-encoding cDNA or empty vector, washed, then incubated with 400 nM synthetic Aß for 6 h. After washing with PBS, intracellular Aß was extracted with 5 M guanidinium isothiocyanate and quantified using a commercially available ELISA (Wako Chemicals USA, Inc.) after 10-fold dilution in the manufacturer-provided dilution buffer.

### Mass spectrometry

The cleavage sites within Aβ40 and Aβ42 hydrolyzed by BACE2 and BACE1 were determined essentially as described [44] with minor modifications. Briefly, Aβ peptides or biotinylated Aβ peptides were incubated for various lengths of time with recombinant BACE2 enzyme in Assay Buffer (25 mM acetate buffer, pH 4.0, supplemented with 0.1% BSA). The reaction was stopped by addition of protease inhibitor cocktail and pH adjustment. Aβ fragments were immediately precipitated by magnetic beads coated with streptavidin (for biotinylated Aβ) or magnetic beads coated with Ab9 antibody [45] (for unmodified Aβ). Beads were washed with 10 mM NH_4_CO_3_, pH 8.0, and peptide fragments were eluted using 0.5% trifluoroacetic acid in 75% acetonitrile in water, followed by the addition of an equal volume of a saturated sinapic acid solution dissolved in 0.5% trifluoroacetic acid in 50% acetonitrile and water. Digested products were spotted onto a gold chip, dried, and analyzed using a Ciphergen ProteinChip SELDI time-of-flight system (Bio-Rad). Mass spectra were acquired automatically in a linear positive mode at 1350 shots per spectrum. Peptides containing a183-Da increase in MW were identified as being modified by AEBSF, as previously reported [46]. Same procedure was applied to detect the endogenous Aβ fragments produced by CHO cells transfected with APP and BACE2 (using Ab9 as a capture antibody).

### In vitro analyses of Aβ degradation by BACE2

The kinetics of Aβ40 and Aβ42 degradation by BACE2 were determined using freshly prepared, monomeric Aβ peptides separated from aggregated species by size-exclusion chromatography and characterized as described [25,26]. Aβ peptides were diluted in neutral Dilution Buffer (20 mM Tris, pH 8.0 supplemented with 0.1% BSA) and reactions were initiated by transfer into 20 times more volume of Assay Buffer supplemented with purified recombinant BACE2 (R&D Systems, nominal concentration 1 or 5 nM) or, as a control for non-specific loss of Aβ, the latter buffer lacking BACE2. Where required, reactions were terminated by supplementation with protease inhibitor cocktail and adjustment to neutral pH. For ELISA-based experiments, Aβ42 and Aβ40 were quantified using the sandwich ELISAs described above. For determination of kinetic parameters, ELISAs were used to quantify the initial velocities of degradation of a range of different concentrations of Aß40 (0.2 to 16 μM) or Aß42 (0.6 to 16 μM) by a fixed amount of recombinant BACE2 (5 nM) in Assay Buffer, and *K*_M_ and *v*_max_ values were determined in triplicate by fitting a hyperbolic curve to these data in Prism 5.0 (GraphPad Software, Inc.). For determination of the pH dependence of Aβ degradation, experiments were carried same as described above, using Assay Buffer at different pH values (3.0, 3.5, 4.0, 4.5, 5.0, 5.5). The reactions were stopped at 10 min and the remaining 200nM of Aβ was determined using a well-characterized fluorescence polarization-based activity Aß degradation assay, as described [23]. For comparison of the rate of degradation of Aβ by different proteases, we incubated 200 nM of Aβ fluorescent substrate (FAβB) with 5nM of different protease in their corresponding buffers: BACE1 and BACE2 using Assay Buffer and IDE, NEP, and plasmin in PBS, pH 7.4 supplemented with 0.1% BSA. The reactions were stopped by addition of protease inhibitor cocktail, 500nM streptavidin, and adjustment to neutral pH. The degree of Aβ hydrolysis was immediately determined using a polarization-based Aß degradation assay [23]. Recombinant BACE2 (R&D Systems) and plasmin (EMD Biosciences) were purchased from commercial sources, while recombinant IDE and secreted NEP (i.e., lacking the transmembrane domain) were generated and purified as described [23]. All reactions were performed at 37°C.

### Fluorescence microscopy

CHO cells (10^6^ cells/cm^2^) were plated onto 8-well poly-D-lysine-coated, glass-bottom chambers (MatTek Corp.) in culture medium (DMEM/Opti-MEM supplemented with 5%FBS). For BACE2 transfections, cell were transfected with a construct encoding BACE2 tagged at its C-terminus with GFP (OriGene Technologies, Inc. Cat. No. RG04860) using Fugene 6.0 transfection reagent according to manufacturer’s recommendations (Promega Corp.). For Aβ colocalization experiments, cells were washed twice in fresh culture medium, then incubated in the latter medium supplemented with either Aß40 (500 nM) labeled at the N-terminus with HiLight Fluor^TM^ 488 or HiLight Fluor^TM^ 555 (AnaSpec, Inc.). For lysosomal staining, cells were incubated with Lysotracker Red according to manufacturer’s recommendations (Invitrogen Corp.), then washed 2 times with fresh culture medium prior to imaging. For confocal microscopy, cells were washed with fresh medium then imaged immediately using the 488-nm and 543-nm laser lines on a Zeiss LCM 510 META confocal microscope (Carl Zeiss, Inc.). Images were processed and analyzed using MetaMorph software according to manufacturer’s recommendations (Molecular Devices, Inc.). For conventional fluorescence microscopy of intracellular Aß, cells were washed with fresh medium, then incubated at 37°C for 1 h prior to imaging using a Nikon Labophot 2 fluorescent microscope (Nikon Inc.).

## Abbreviations

Aß: Amyloid ß-protein; AßDP: Aß-degrading protease; AD: Alzheimer disease; AP: Alkaline phosphatase; APP: Amyloid precursor protein; BACE1: ß-site APP-cleaving enzyme-1; BACE2: ß-site APP-cleaving enzyme-2; ECE1: Endothelin-converting enzyme-1; IDE: Insulin-degrading enzyme; NEP: Neprilysin.

## Competing interests

The authors declare they have no competing interests.

## Authors' contributions

SA-H contributed to the design of experiments, executed the screen and all follow up experiments, analyzed data, and drafted the manuscript. TS assisted with the execution of the primary screen. MM and DK assisted with the maintenance of cell cultures. ML conceived of the experimental approach, designed experiments, analyzed data and wrote the manuscript. All authors read and approved the final manuscript.
